# Modeling the Transmission of Foot and Mouth Disease to Inform Transportation of Infected Carcasses to a Disposal Site During an Outbreak Event

**DOI:** 10.3389/fvets.2019.00501

**Published:** 2020-01-14

**Authors:** Emily Walz, Jamie Middleton, Fernando Sampedro, Kimberly VanderWaal, Sasidhar Malladi, Timothy Goldsmith

**Affiliations:** ^1^Department of Veterinary and Biomedical Sciences, College of Veterinary Medicine, University of Minnesota, St. Paul, MN, United States; ^2^Center for Animal Health and Food Safety, College of Veterinary Medicine, University of Minnesota, St. Paul, MN, United States; ^3^Environmental Health Sciences Division, School of Public Health, University of Minnesota, Minneapolis, MN, United States; ^4^Department of Veterinary Population Medicine, College of Veterinary Medicine, University of Minnesota, St. Paul, MN, United States

**Keywords:** foot and mouth disease, FMDv, carcass, cattle, swine

## Abstract

In the event of a Food and Mouth Disease (FMD) outbreak in the United States, an infected livestock premises is likely to result in a high number of carcasses (swine and/or cattle) as a result of depopulation. If relocating infected carcasses to an off-site disposal site is allowed, the virus may have increased opportunity to spread to uninfected premises and result in exposure of susceptible livestock. A stochastic within-herd disease spread model was used to predict the time to detect the disease by observation of clinical signs within the herd, and the number of animals in different disease stages over time. Expert opinion was elicited to estimate depopulation parameters in various scenarios. Disease detection was assumed when 5% of the population showed clinical signs by direct observation. Time to detection (5 and 95th percentile values) was estimated for all swine farm sizes (500–10,000 head) ranged from 102 to 282 h, from 42 to 216 h for all dairy cattle premises sizes (100–2,000 head) and from 66 to 240 h for all beef cattle premises sizes (5,000–50,000 head). Total time from infection to beginning depopulation (including disease detection and confirmation) for the first FMD infected case was estimated between 8.5–14.3 days for swine, 6–12.8 days for dairy or beef cattle premises. Total time estimated for subsequent FMD cases was between 6.8–12.3 days for swine, 4.3–10.8 days for dairy and 4.5–10.5 days for beef cattle premises. On an average sized operation, a sizable proportion of animals in the herd (34–56% of swine, 48–60% of dairy cattle, and 47–60% of beef cattle for the first case and 49–60% of swine, 55–60% of dairy cattle, 56–59% of beef cattle for subsequent cases) would be viremic at the time of beginning depopulation. A very small fraction of body fluids from the carcasses (i.e., 1 mL) would contain virus that greatly exceeds the minimum infectious dose by oral (4–7x) or inhalation (7–13x) route for pigs and cattle.

## Introduction

Foot and mouth disease (FMD) is a highly contagious viral disease affecting primarily cloven-hoofed animal including key livestock production species such as cattle and swine. In the event that a case of FMD were detected in the United States (US), there would likely be serious economic impact on international trade of animals and animal products (1). The US has a preparedness and response plan for disease control and eradication in the event of a foreign animal disease event. This plan encompasses management of multiple animal species, and may include movement control, quarantine, vaccination, and depopulation measures (2).

Identification of FMD within a herd relies upon observation of clinical signs to trigger diagnostic testing of suspect individuals. Testing methods for population-level disease surveillance are lacking; this likely results in delayed detection until infection has spread at the farm level. Experimental and modeling studies of transmission in cattle (3) and swine (4) suggest that the infectious period in these species as close as under 24 h before the onset of clinical signs (fever or lesions). This underscores the important role of prompt detection by clinical signs to limit spread throughout the herd. Similarly, early detection decreased the length of epidemics in a multi-species model based on a cattle and feedlot-dense region of Texas, USA (5).

Depopulating an infected premises is performed to prevent further spread of Foot and Mouth Disease virus (FMDv) to susceptible animals and to limit additional FMDv shedding in latently or clinically infected individuals. If an outbreak were concentrated in a geographic area in which FMD can be readily contained without further spread, the response strategy of “stamping out” will likely be elected. “Stamping out,” or immediate depopulation, is the preferred control method for clinically infected and in-contact susceptible animals as a means to reduce the potential of disease spread. It is assumed that the depopulation procedures would follow the United States Department of Agriculture, Foreign Animal Disease Preparedness and Response Plan (FAD PReP) Guidance (2).

FMD was eradicated from the United States in 1929 (6); historical data specific to the modern large-scale agricultural operations most common in the US are lacking. Especially in areas where empirical data is lacking, expert opinion has been a mainstay in informing proactive planning for FMD incursions, and aspects such as disease characteristics in a naïve population and depopulation techniques have been described (7, 8). Models have been used as another means of understanding potential disease scenarios and as a way to inform planning decisions. Most models focus on between-herd spread, and incorporate aspects such as vaccination strategies, movement characteristics, and geographic proximity in areas with multiple species, such as cattle, goats, and swine (5, 9–12). In all these studies depopulation is one option to limit disease spread; however, management strategies for carcasses after depopulation was not considered.

**Figure 1:**
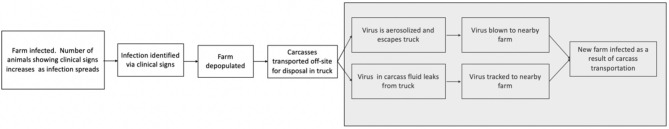
Disease spread pathway by which FMDv may spread during the transportation of carcasses from an infected livestock premises to an off-site disposal location. Steps in gray represent the potential transmisson risks during transportation.

Swine and cattle (beef and dairy) are the two most prevalent livestock species in the US (13, 14). If a swine or cattle premises were infected and depopulated, the option to dispose of carcasses off-site may be needed due to environmental and other limitations of disposing a large biomass on-site. It is required for trucks to be leak-proof while hauling animal carcasses according to US Code of Federal Regulations (15), however, in the event of an FMD outbreak, other means of hauling carcasses may be employed. FMDv presents a containment challenge due to its persistence in the environment, especially when it is within organic material and protected from desiccation, heat and adverse pH conditions (16). Movement of FMDv-infected carcasses represents one of the main disease spread pathways during an outbreak. Proactively evaulating the potential risk of transmission and available mitigation meaures can allow risk managers to be better prepared for these scenarios in the event of an outbreak (Figure 1).

The aim of this study was to evaluate the likelihood that carcasses in a truckload from a depopulated infected premises would contain an infective FMDv dose at the time of transportation to disposal. This information is an important consideration for emergency preparedness and management officials in the event of a FMD outbreak, as off-site transportation of carcasses to disposal is a potential pathway to spread virus during an outbreak.

## Materials and Methods

A stochastic disease spread model was developed to simulate the transmission of FMDv within a swine, dairy or beef cattle herd and predict the proportion of viremic animals at the time of depopulation. The model was run for each of the livestock types, and it estimated the number of animals in various disease stages at each time step. Disease stages included: susceptible (S), latent (L), pre-clinical (PI), clinical (CI), and recovered (R) (17). Both pre-clinical and clinical animals were considered viremic and infectious to other susceptible animals within the herd (18, 19). The model updated the number of animals in each disease state every 6 h. The uncertainties in input variables, as well as the inherent variability associated with the course of infection in individual swine, dairy and beef cattle populations and the spread within the group were considered in the model in the form of distributions for the different parameters (transmission coefficient, duration of the latent, pre-clinical and clinical periods). Parameter distributions were obtained from previous FMD modeling studies and meta-analyses (18–20). The farm size scenarios used in the model were based on a compilation of statistics published by the National Agricultural Statistics Service (NASS) of the United States Department of Agriculture for 2014 (21). Average farm sizes were calculated for all production types within livestock category (swine, beef cattle, or dairy cattle). The model assumed that disease transmission was the same regardless of animal age. Table 1 shows the inputs used in the disease spread model.

**Table 1:** Input parameters used in the FMD spread model in swine, dairy and beef cattle premises.

**Variable^a^**	**Input distribution/value**	**References**
Latent period	Normal (2.31, 1.40) (swine) Weibull (1.78, 3.97) (cattle)	(19) (18)
Pre-clinical period	Normal (1.485, 1.099) (swine) Gamma (α = 1.222, θ = 1.672) (cattle)	(19) (18)
Clinical period	Poisson (λ = 5.195)-Normal (1.485, 1.099) (swine)	(19)
	Gamma (α = 4.752, θ = 0.736) (cattle)	(20)
Group size (head)	500, 1,000, 5,000, and 10,000 (swine) 100, 500, 1,000, and 2,000 (dairy) 5,000, 15,000, 30,000, and 50,000 (beef)	(21)
Adequate contact rate (contacts/day)	beta PERT (3.17, 6.84, 14) (swine)	(22)
	beta PERT (13, 54, 216) (cattle)	(17)
Detection threshold	5% of group	

a *Distributions refer to swine groups of more than 200 head*.

The model assumed random mixing among the entire population. The number of susceptible animals that become infected in each time step in the model was dependent on the adequate contact rate and the proportion of infectious animals in the herd at that time step. The same contact rate was used for both index and subsequent case scenarios. The adequate contact rate (*k*) is defined as the mean number of other animals each infected animal comes into contact with per unit time such that the contact is adequate to transmit infection. Thus, the probability (*P*_*t*_*)* that an animal becomes infected and the number of newly infected, latent individuals ($L_{t+1}^{new}$) in a given time step can be expressed as:

(1) $$\begin{array}{r} P_{t}=1-e^{-\left(\frac{k{\;I}_{t}}{N-1}\right)} \end{array}$$

(2) $$\begin{array}{r} L_{t+1}^{new}\sim Binomial\left(S_{t},P_{t}\right) \end{array}$$

where *N* is the total population size of the farm, *I*_*t*_ is the number of infectious animals (pre-clinical or clinical) and *S*_*t*_ is the number of susceptible individuals at time *t*. Transitions between other disease stages (from L to PI, PI to CI and CI to R disease stages) were simulated based on the duration of each period, which was determined individually for each animal.

The disease spread model also estimated the time to detect FMD infection in the herd based on the active observation of clinical signs, which is one of the surveillance measures that may be applied in an outbreak at the herd level (23). The threshold for detection of the disease was set at 5% of the herd showing clinical signs, which was based on the percentage of naturally occurring lameness on swine and cattle farms (24, 25). A sensitivity analysis on the detection threshold (re-analyzed at 2.5 or 10%) was performed for an exemplar scenario (swine herd of 5,000 head) to ensure that time to detection distributions were not overly sensitive to changes in this threshold (Supplementary Figure 1).

Once the disease was detected at a premises, it was assumed that a depopulation protocol would be initiated by disease management officials. Total time from detection to beginning depopulation was estimated by adding each time interval by using the following equation:

(3) $$\text{Total time}={\text{t}}_{\det\;}+\;{\text{t}}_{\text{conf }}+\;{\text{t}}_{\text{sdep}}(\text{h})$$

where, t_det_ is the time elapsed to detect FMD post-infection depending on the farm size, t_conf_ is the time interval between detection of clinical signs in a particular premises to the official laboratory confirmation of a positive sample, and t_sdep_ is the time interval between laboratory confirmation to starting depopulation. All the time intervals were expressed in hours.

Expert opinion was solicited via email from five national experts in emergency management and depopulation procedures working in academia, industry and government settings to provide estimates on time intervals for laboratory confirmation after the detection of an infected premises and for starting the depopulation protocol (Supplementary Table 1). It was assumed that the time to complete indemnity or time to find disposal options were not included in the estimation of total time from infection to depopulation. Two scenarios (index case and subsequent cases) were given to the experts for estimating the time to start the depopulation procedure (t_sdep_). Input values of equation 3 for swine, dairy and beef cattle as the index case are shown in Tables 2–4. A Pert distribution was used to characterize the variability among experts' responses (26). The worst-case scenario was selected to populate the distribution by identifying the longest time interval estimates among all the experts for the minimum, most likely and maximum values. For subsequent cases, the time from disease detection to laboratory confirmation and the time from confirmation to beginning depopulation were each set at 24 h. A Monte Carlo simulation was carried out by using @Risk 6.2 for Excel (Palisade Corporation, NY). The analysis was performed using 1,000 iterations with Latin-hypercube method. Outputs were expressed by the mean and 90% prediction intervals as calculated by the 5th and 95th percentile values. The proportion of viremic and recovered animals at the time of starting depopulation was predicted from the disease transmission model at the time elapsed between infection and starting the depopulation.

**Table 2:** Input values to estimate timings for depopulation procedure in case of FMD outbreak in swine premises.

	**Stochastic disease spread model**	**Expert elicitation**		
**Herd size**	Time to detect disease post-infection (h)^*^	**Time from disease detection to laboratory confirmation (h)**	**Time from confirmation to starting depopulation (h)**	Depopulation rate (head/h)^**^
500	PERT (102, 135, 228)	PERT (24, 48, 72)	PERT (24, 48, 72)	PERT (30, 140, 600)
2,000	PERT (120, 155, 246)			
5,000	PERT (132, 172, 270)			
10,000	PERT (144, 183, 282)			

*Beta-PERT distributions represent minimum, most likely, and maximum values*.

* *5% detection level*.

** *Using three crews (8 men each) during 3 working shifts (20 + 4 h cleaning) and two side discharge alleys with two loaders*.

**Table 3:** Input values to estimate timings for depopulation procedure in case of FMD outbreak in dairy premises.

	**Stochastic disease spread model**	**Expert elicitation**		
**Herd size**	Time to detect disease post-infection (h)^*^	**Time from disease detection to laboratory confirmation (h)**	**Time from confirmation to starting depopulation (h)**	Depopulation rate (head/h)^**^
100	PERT (42, 82, 192)	PERT (24, 48, 72)	PERT (24, 48, 72)	PERT (18, 36, 60)
500	PERT (54, 93, 192)			
1,000	PERT (60, 97, 192)			
2,000	PERT (60, 107, 216)			

*Beta-PERT distributions represent minimum, most likely, and maximum values*.

* *5% detection level*.

** *Using three crews (8 men each) during 3 working shifts (20 + 4 h cleaning) and two cow side discharge alleys (10 cows each) with two loaders*.

**Table 4:** Input values to estimate timings for depopulation procedure in case of FMD outbreak in beef cattle premises.

	**Stochastic disease spread model**	**Expert elicitation**		
**Herd size**	Time to detect disease post-infection (h)^*^	**Time from disease detection to laboratory confirmation (h)**	**Time from confirmation to starting depopulation (h)**	Depopulation rate (head/h)^**^
5,000	PERT (66, 109, 216)	PERT (24, 48, 72)	PERT (24, 48, 72)	PERT (18, 36, 60)
15,000	PERT (72, 117, 210)			
30,000	PERT (78, 120, 216)			
50,000	PERT (78, 128, 240)			

*Beta-PERT distributions represent minimum, most likely, and maximum values*.

* *5% detection level*.

** *Using three crews (8 men each) during 3 working shifts (20 h + 4 h cleaning) and two cow side discharge alleys (10 cows each) with two loaders*.

## Results

The disease spread model estimated the time to reach 5% of clinical animals in the herd (threshold for FMD detection by active observational surveillance). Time to detection (5 and 95th percentile values) was estimated at 102–282 h for all swine farm sizes (500–10,000 head), from 42 to 216 h for all dairy cattle premises sizes (100–2,000 head), and from 66 to 240 h for all beef cattle premises sizes (5,000–50,000 head). A sensitivity analysis of the detection threshold demonstrated that the distributions for time to detection were not sensitive to the threshold.

**Figure 2:**
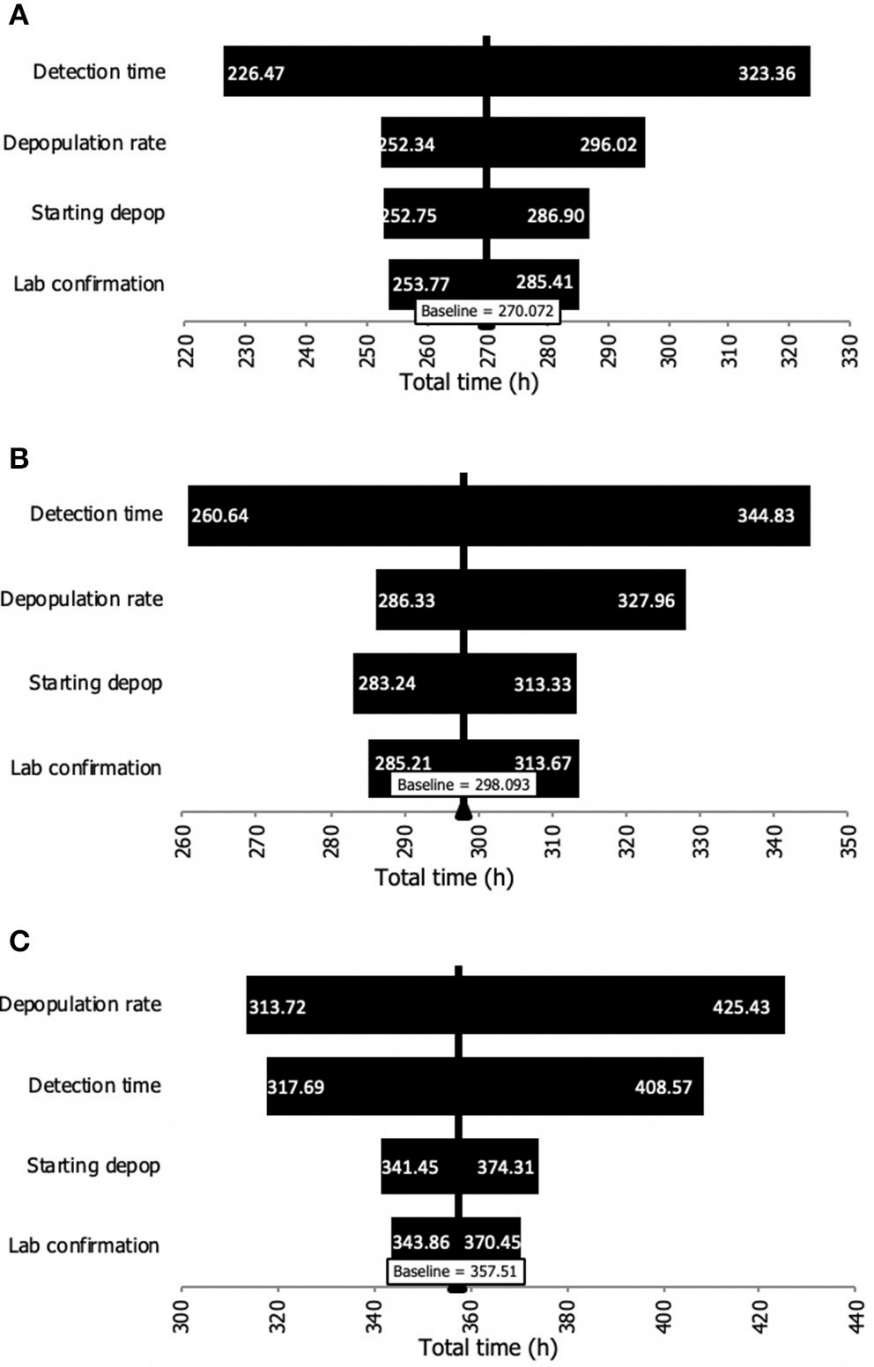
Sensitivity analysis of the influence of the input time intervals on the total time from detection to depopulation of premises during an FMD outbreak. **(A)** Dairy premises. **(B)** Swine premises. **(C)** Beef cattle premises.

A sensitivity analysis was carried out to identify the time interval that had the greatest influence on the total time (from detection to finalized depopulation). As it can be seen in Figure 2, the detection time was the input variable with the greatest influence for dairy and swine premises. However, given the size (i.e., large number of animals on beef premises), the time to depopulate a farm was the interval with the greatest influence on beef premises.

Total time from infection to depopulation (90% prediction interval) for the first FMD infected case was estimated to be 8.5–14.3 days for a 3,000 head swine herd, 6.0–12.8 days for a 2,000 head dairy herd and 6.0–12.8 days for 5,000 head beef cattle premises (Tables 2–4). Total time estimated for subsequent FMD cases is reported in Table 5. A sizable proportion of animals in the herd (34–56% of swine, 48–60% of dairy cattle, and 47–60% of beef cattle for the first case, and 49–60% of swine, 55–60% of dairy cattle, 56–59% of beef cattle for subsequent cases) would be viremic at the time of depopulation (Table 6).

**Table 5:** Total time from infection to beginning depopulation.

**Type of farm**	Total time (days)^*^	
	**First index case**	Subsequent cases^**^
Swine (3,000 head)	10.8 (8.5–14.3)	8.8 (6.8–12.3)
Dairy (2,000 head)	8.5 (6.0–12.8)	6.5 (4.3–10.8)
Beef cattle (5,000 head)	8.6 (6.0–12.8)	6.6 (4.5–10.5)

* *1,000 iterations (mean, 5th and 95th percentile values)*.

** *Time from detection (including disease confirmation) to starting depopulation was set at 48 h for subsequent FMD cases*.

**Table 6:** Number of viremic and recovered animals at the start of depopulation.

	**First index case**		
	Time elapsed before depopulation (days)^*^	**Percentage of viremic animals (pre-clinical + clinical) (%)**	**Percentage of recovered animals (%)**
Swine (3,000 head)	10.8 (8.5–14.3)	46 (34–56)	46 (28–62)
Dairy cattle (2,000 head)	8.5 (6.0–12.8)	55 (48–60)	34 (22–45)
Feedlot cattle (5,000 head)	8.6 (6.0–12.8)	55 (47–60)	37 (22–47)
	Subsequent cases^**^		
Swine (3,000 head)	8.8 (6.8–12.3)	56 (49–60)	15 (13–18)
Dairy cattle (2,000 head)	6.5 (4.3–10.8)	57 (55–60)	12 (10–14)
Feedlot cattle (5,000 head)	6.6 (4.5–10.5)	58 (56–59)	12 (10–14)

* 1,000 iterations. Reported values represent mean, 5th and 95th percentile values

** *Time from detection (including disease confirmation) to starting depopulation was set at 48 h for subsequent FMD cases*.

## Discussion

Model outputs suggest that if a herd is depopulated when 5% of animals show active clinical signs, a large proportion of the herd will be viremic at the time of beginning depopulation. Even in subsequent cases where it is assumed that the time to from disease detection to depopulation will be shorter (48 h), the proportion of viremic animals remains relatively unchanged. Moving infected carcasses represents a real risk for FMDv spread during an outbreak. However, in the event that “stamping out” is employed, off-site disposal is likely to be required due to the size of beef, dairy, and commercial swine premises in the US and the large amount biomass resulting from depopulation.

Virus could escape from a load of carcasses in leaked fluid, expelled fomites (e.g., dirt, feces), or jostled carcasses from the load, or via aerosolization of virus-laden particulate matter. The likelihood of a spill or aerosol event is unknown, however it is likely that even a small volume of escaped fluid may contain an infectious dose of virus. The average concentration of FMDv in a carcass in experimental inoculation studies was 10^3^ PFU/g for a pig carcass and 10^6^ PFU/g for a cattle carcass (27–40). Consultation with rendering industry experts revealed that for transportation of fresh, intact carcasses under normal conditions, most body fluids remain inside the carcass (personal communication, 2013). In a full load of a standard rendering truck (29–1,000 carcasses), experts estimated the amount of fluid leakage from carcasses at 20 L per load. Assuming that 1 mL of leakage contains equivalent virus to 1 g of carcass material, 1 mL of body fluids could contain 10–100,000 times higher virus quantity (10^3^-10^6^ PFU) than the minimum infectious dose by oral (1.4 × 10^4^ – 1.4 × 10^6^ PFU) and inhalation route (7–357 PFU) for pigs and cattle (41, 42). Of note, these estimates are based on literature review and experimental studies; virus loads in tissues may be different among virus strains and subtypes or in non-experimental conditions, however, this data was not available for extrapolation.

The environmental conditions which favor airborne FMDv spread are high humidity, low precipitation, low to moderate wind speed, and flat terrain (43). Suitable conditions of relative humidity (RH) above 60% and temperatures below 33°C (91°F) are needed for long-range airborne transmission to be possible. FMDv bioaerosols degrade quickly in RH below 55% due to desiccation (44). Precipitation generally reduces atmospheric bioaerosol concentrations, while high levels of turbulence temporarily increase aerosolized concentrations when dust is raised (45). In longer-range airflows, turbulence eventually causes dilution of FMD bioaerosol concentrations and higher gravitational sedimentation, especially in particles smaller than 10 micrometers (46). Sunlight has minimal effect on the aerosol spread of FMDv, and instead mostly affects survival on surfaces (46). While beyond the scope of this study, further work on the risks of aerosol spread may be warranted if off-site carcass transportation is considered.

A standard rendering truck is outfitted with sealed tailgate and tarp cover to prevent spills or aerosolization, however, it is unlikely that this will completely mitigate risk of virus escape from a load. In the event of an outbreak, other truck types may be employed due to increased demand for timely carcass disposal. A standard rendering truck, roll-off, or dump truck without tarp covering would have an increased likelihood of spillage, due to the proximity of carcasses and other contaminated debris to the top of the trailer in a full load. The use of a sealed plastic bag suitable for the disposal of biological residues is an option provide full protection against spillage and aerosolization. In the event that new or different types of equipment are employed, or that new personnel lacking adequate training are used during an outbreak, the potential that standard mitigation measures may be misused due to human error cannot be underestimated.

Due to the proactive nature of this assessment, some assumptions in calculations were made which may limit this model's applicability in the event of an outbreak. For example, in estimating the time until FMD detection on a farm, only direct animal-animal contact was considered in disease spread, however, in some geographies or production systems, aerosol or fomite (contaminated person/equipment) may also contribute to spread. In addition, the presence of segregated or sub-herds within a population would change the contact rate and the number of animals with viremia at different time points. However, an analysis by Kinsley showed that adding within-farm population structure did not substantially influence time to detection or time to the peak of the epidemic (47). Additionally, although a change in time to detection (either shorter or longer) could influence our results, we did not find time to detection to be influenced by the detection threshold (percent of animals clinical). Part of the reason for this is the high transmission rate of the virus. By the time 5% of the animal are clinical, transmission is in its exponential growth phase (48), and the difference between time until 5 vs. 10% are clinically infected is very small. In addition, our results for time to detection, derived from the stochastic model, were consistent with an analysis of real-world data from the UK epidemic, where the probability of a farm escaping detection fell sharply at around 7 days and was negligible by 12–13 days (49).

In the event of an especially large infected premises, such as a feedlot operation or an integrated farrow to finish swine operation, depopulation (even at efficient speeds) may last weeks to months. The proportion of viremic animals near the end of a depopulation effort and after significant time has elapsed in disease progression is likely markedly different than that which was calculated at the start of depopulation. Further modeling of this disease progression is an area for further work which may be instrumental in planning for management of large infected premises.

In calculating length of time to depopulation, it was assumed that the disposal site was identified and secured before the outbreak, and no additional delays in depopulation or transportation of carcasses occurred as a result of having to locate an acceptable disposal site. It was assumed that the time from depopulation to movement of carcasses to the disposal site would be very short (a matter of hours), so the potential for body fluids to escape from carcasses (leakage) will be minimized. In the event that depopulation or movement of carcasses from euthanasia location into transport vehicle is delayed, it is likely that larger amounts of body fluids may be present, and risks associated with leakage from carcasses may become more significant. Additional delays in transportation or increased duration of transportation to distant disposal sites can be expected to have similar effects on increased leakage as additional body fluids and products of autolysis escape from a carcass.

Finally, this study did not consider issues related to capacity, resource availability, and resource depletion. A large number of infected premises over an extended time period would have the potential to deplete available resources as well as capacity. This would likely result in longer delays in identification, depopulation and disposal. In this event, the herds continue to progress toward a recovered stage, and the proportion of the herd which is viremic will continue to decrease, while the potential for viral contamination of the premises will increase. Issues of logistics and animal welfare must be balanced with the potential for depopulation to decrease the number of potential animal hosts in a local area.

## Conclusion

In the event of an FMD outbreak in the US, significant time will lapse between infection of a livestock premises and beginning depopulation. During this time, disease continues to spread throughout a herd, and it is likely that a large proportion of animals will be viremic at the time of depopulation, even if disease confirmation and beginning depopulation occurs in a timely manner. Given that even a small amount of leakage from viremic carcasses is likely to contain FMDv concentrations that will exceed the minimum FMD infective dose for pigs and cattle by several degrees of magnitude, it appears that leakage from vehicles transporting viremic carcasses to off-site disposal locations represent a real risk for virus spread during an outbreak. Delays in identification, depopulation and disposal will likely result in greater number of animals that are in the recovered stage.

This study can inform the risk assessment of FMD transmission during the movement of infected carcasses, and should be valuable for risk managers when considering emergency response options. In addition, this can help federal and state agencies to adopt additional risk mitigation measures to reduce the likelihood of infection of susceptible livestock during an FMD outbreak in the US.

## Data Availability Statement

All datasets generated for this study are included in the article/Supplementary Material.

## Author Contributions

TG and FS led project development and provided oversight for this work. FS, SM, and KV developed the modeling components for this study. JM participated in drafting initial reports and literature review. EW updated and synthesized previous work and wrote the manuscript. All authors reviewed and provided critical feedback on the manuscript before submission.

### Conflict of Interest

The authors declare that the research was conducted in the absence of any commercial or financial relationships that could be construed as a potential conflict of interest.
