# Supplementary material for: Modeling the Transmission of Foot and Mouth Disease to Inform Transportation of Infected Carcasses to a Disposal Site During an Outbreak Event
Source: Front Vet Sci. 2020 Jan 14;6:501. doi: 10.3389/fvets.2019.00501 (PMC6971117; doi:10.3389/fvets.2019.00501)
Supplement: Supplementary file 1 [file Table_1.DOCX]

Supplementary Table 1. Results of expert opinion solicited via email from five (U.S-based) national experts in emergency management and depopulation procedures. Expert responses assume that the outbreak is localized, and all the needed resources are readily available.

| Expert | Time to start depopulation (h) | Depopulation time (heads/h)^a^ |
| --- | --- | --- |
| Expert A | Min: 24  Most likely: 48  Max: 72 | Min: 30  Most likely: 50  Max: 75 |
| Expert B | Min: 12  Most likely: 48  Max: 72 | Min: 4*3 crews=12 (cattle)  20*3 crews=60 (swine)  Most likely: 8*3 crews=24 (cattle)  160*3 crews=480 (swine)  Max: 300*3 crews=900 (swine) |
| Expert C | Min: 12  Most likely: 24  Max: 48 | Min: 6*3 crews=18  Most likely: 12*3 crews=36  Max: 20*3 crews=60 |
| Expert D | Min: 24  Most likely: 36  Max: 48 |  |
| Expert E | Min: 24  Most likely: 36  Max: 60 | Cash pistol-grip captive bolt: 30  CO2 gassing roll-off trailers: 140  Mobile electrocution trailer: 600  Maximum rendering capacity: 167 |

^a^ Using three eight-person crews (20 h work +4 h cleaning) and two ten-cow side discharge alleys with two loaders
