# Supplementary material for: Modeling the Transmission of Foot and Mouth Disease to Inform Transportation of Infected Carcasses to a Disposal Site During an Outbreak Event
Source: Front Vet Sci. 2020 Jan 14;6:501. doi: 10.3389/fvets.2019.00501 (PMC6971117; doi:10.3389/fvets.2019.00501)
Supplement: Supplementary file 2 [file Image_1.pdf]

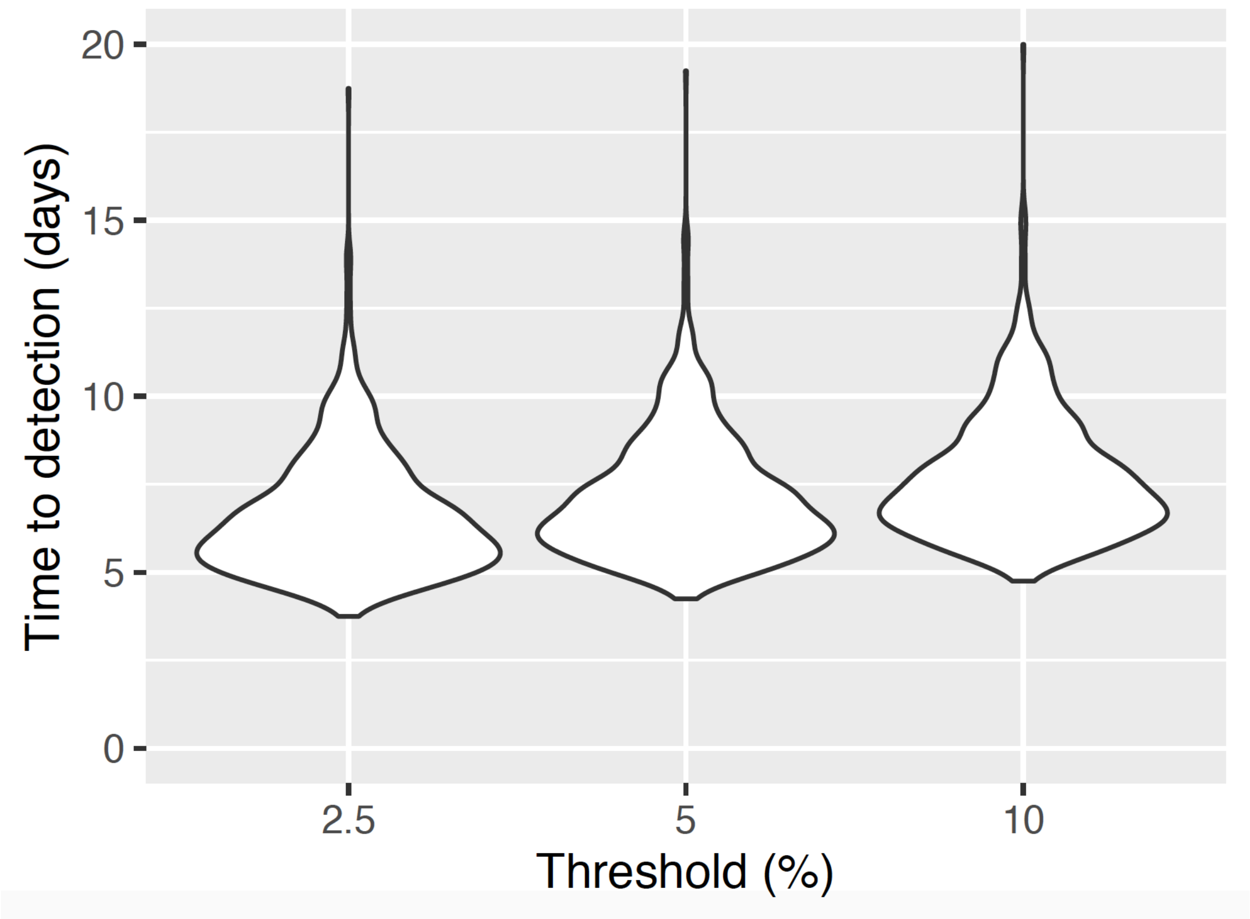

Supplementary Figure 1. Results of a sensitivity analysis on the detection threshold (analyzed at 2.5%, 5% or 10%) was performed for an exemplar scenario (swine herd of 5,000 head) to ensure that time to detection distributions were not overly sensitive to changes in this threshold.
